# Inflammasome pathway in kidney transplantation

**DOI:** 10.3389/fmed.2023.1303110

**Published:** 2023-11-08

**Authors:** Simona Granata, Daniele La Russa, Giovanni Stallone, Anna Perri, Gianluigi Zaza

**Affiliations:** ^1^Nephrology, Dialysis and Transplantation Unit, Department of Medical and Surgical Sciences, University of Foggia, Foggia, Italy; ^2^Department of Biology, Ecology and Earth Sciences, University of Calabria, Rende, Italy; ^3^Department of Experimental and Clinical Medicine, University of Catanzaro "Magna Græcia", Catanzaro, Italy

**Keywords:** inflammasome, kidney transplantation, NLRP3, post-transplant complications, immune response

## Abstract

Kidney transplantation is the best available renal replacement therapy for patients with end-stage kidney disease and is associated with better quality of life and patient survival compared with dialysis. However, despite the significant technical and pharmaceutical advances in this field, kidney transplant recipients are still characterized by reduced long-term graft survival. In fact, almost half of the patients lose their allograft after 15–20 years. Most of the conditions leading to graft loss are triggered by the activation of a large immune-inflammatory machinery. In this context, several inflammatory markers have been identified, and the deregulation of the inflammasome (NLRP3, NLRP1, NLRC4, AIM2), a multiprotein complex activated by either whole pathogens (including fungi, bacteria, and viruses) or host-derived molecules, seems to play a pivotal pathogenetic role. However, the biological mechanisms leading to inflammasome activation in patients developing post-transplant complications (including, ischemia-reperfusion injury, rejections, infections) are still largely unrecognized, and only a few research reports, reviewed in this manuscript, have addressed the association between abnormal activation of this pathway and the onset/development of major clinical effects. Finally, the regulation of the inflammasome machinery could represent in future a valuable therapeutic target in kidney transplantation.

## 1. Introduction

Kidney transplantation is the best treatment for patients with end-stage kidney disease (ESKD), a clinical condition characterized by severe alterations in body homeostasis that require the initiation of renal replacement therapy to ensure patient survival. It is associated with a better quality of life and survival compared with dialysis treatment (1).

In addition, during the last two decades, the significant advancement of surgical and organ preservation techniques and the optimization of immunosuppressive drug management has led to an important improvement in short-term graft survival (more than 90% after 1 year) (2). However, despite this clinical success, the long-term allograft outcome remains unsatisfactory. The 10-year graft survival rate is 65.5% among living donor transplant recipients and 49.5% among kidney transplant recipients from deceased donors (3).

Several demographic and clinical factors have been considered responsible for this condition (e.g., donor/recipient age, marginality of the kidney, delayed graft function (DGF), acute rejection, infections, and glomerulonephritis recurrence), but the entire biological machinery involved in graft loss is still only partially defined.

Numerous biological elements have been associated with pathogenetic alterations leading to post-transplant complications, but inflammation seems to play a pivotal role in almost all clinical allograft alterations. In addition, several reports have recently suggested that the inflammasome, a multiprotein complex activated by either whole pathogens (including fungi, bacteria, and viruses) or host-derived molecules as in chronic kidney disease (4–6), could be involved in these post-transplant complications (7).

**Figure 1:**
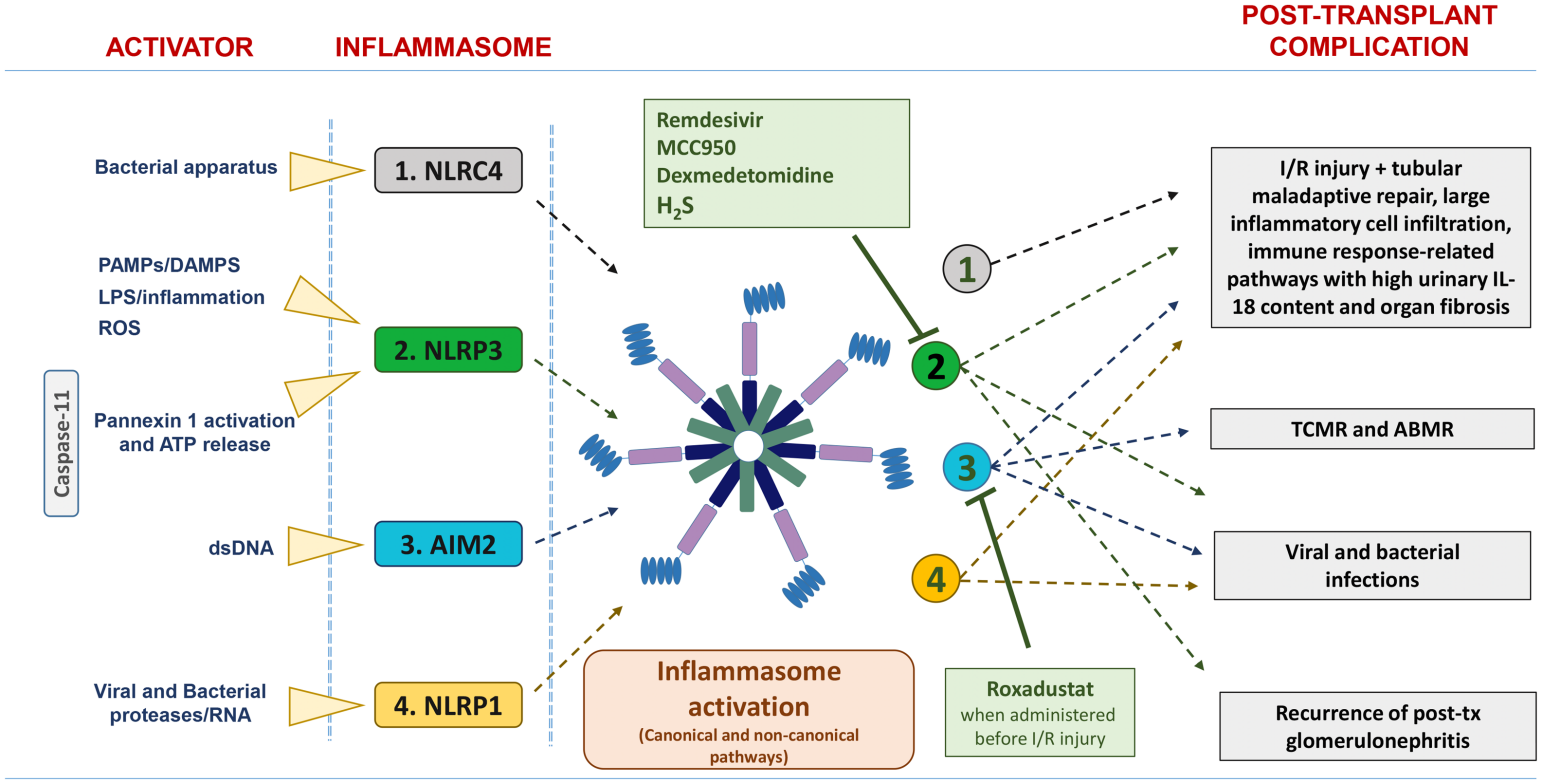
List of the main activators and inflammasomes involved in post-transplant complications. NLRC4, NLRP3, and AIM2 are activated in IRI/DGF, and their persistent over-expression was associated with tubular maladaptive repair, large inflammatory cell infiltration, and organ fibrosis. Up-regulation of AIM2 inflammasome has been found in acute rejection, particularly TCMR. NLRP3, AIM2, and NLRP1 are involved in viral and bacterial infection. The pharmacological inhibitors Remdesivir, MCC950, dexmedetomidine, and H2S suppress NLRP3 inflammasome activation, whereas Roxadustat downregulates AIM2 inflammasome activation.

In this review, which focuses only on kidney transplantation, we provide a brief overview of the potential role of inflammasomes in the pathogenesis of major acute and chronic allograft complications (including ischemia/reperfusion injury, graft rejections, post-transplant infections and recurrent glomerulonephritis) (Figure 1).

## 2. Structure and molecular components of the inflammasome

The inflammasome is a multiprotein complex comprising three classes of molecules: receptor/sensor, adaptor, and effector. Inflammasome sensors (or pattern recognition receptors, PRR) belong to two classes: nucleotide oligomerization domain (NOD)-like receptors (NLRs), and absent in melanoma-2 (AIM2)-like receptors (ALRs) (8). The adaptor molecule used in most inflammasomes is apoptosis-associated speck-like protein (ASC) that enables the recruitment of pro-caspase 1, an effector molecule that mediates maturation of pro-interleukin (IL)-1β and pro-IL-18 to fully active interleukins that are crucial for host defense responses to infection and injury (9). In addition, inflammasomes trigger the pyroptosis (10) a programmed pro-inflammatory cell death mediated by gasdermin D (GSDMD), which forms pores in the plasma membrane, inducing the release of IL-1β and IL-18, water influx, cell swelling, and consequent osmotic lysis (11–14).

The assembly of inflammasomes begins with the activation of PRRs (15, 16). These molecules are capable of recognizing various pathogen-associated molecular patterns (PAMPs) (such as lipopolysaccharide (LPS) and flagellin) and danger-associated molecular patterns (DAMPs) such as uric acid, ATP, DNA, and High Mobility Group 1 (HMGB1) which arise during microbial infections and sterile injury, respectively (15).

The human genome encodes for 23 NLRs; however, only a few NLR proteins, such as NLRP1, NLRP3, NLRP6, NLRP7, NLRP9, NLRP12, and NLRC4, have been found to form inflammasomes and activate caspase-1 (8). These NLRs share some similar structures: C-terminal leucine-rich repeat (LRR) domains, which are responsible for ligand sensing, and central nucleotide binding domains (NACHT), which elicit ATP-induced oligomerization. The N-terminal region contains the pyrin domain (PYD) in NLRP and one or more caspase recruitment (CARD) domains in NLRC proteins (17, 18).

The AIM2 protein consists of two domains: (1) a C-terminal HIN domain, which allows for the detection of double-stranded DNA in the cytoplasm, and (2) a PYD domain for the recruitment of ASCs and formation of the inflammasome complex (19, 20).

NLRP1, NLRP3, NLRP6, NLRP7, NLRP9, NLRP12, and NLRC4 belong to the group of “canonical inflammasomes” that induce the activation of IL-1β and IL-18 through caspase-1, whereas caspase-11 (in mouse) and caspase-4 and -5 (in humans) are defined “non-canonical” (21). These non-canonical inflammasomes consist of three main domains: an N-terminal CARD, a p20, and a C-terminal p10 (22). Although activated by different types of PAMPs and DAMPs, they have similar downstream effector functions, including the activation of GSDMD-mediated pyroptosis and the activation of caspase-1, leading to subsequent proteolytic maturation of IL-1β and IL-18 and their secretion through GSDMD-mediated pores (23–26).

Among the canonical inflammasomes, NLRP3 is the most studied, being largely involved in both innate and adaptive immunity after activation by either whole pathogens, including fungi, bacteria, and viruses, or by host-derived molecules, such as fibrillar amyloid-β (Aβ) peptide, as well as extracellular ATP and glucose (27–31).

The NLRP3 inflammasome may be activated by both canonical and noncanonical pathways.

## 3. The canonical activation of NLRP3 inflammasome

This pathway consists of two steps: priming and activation. Priming signals include microbial components or endogenous cytokines that bind to the Toll-like receptor (TLR), FAS-associated death domain protein (FADD) or IL-1R ligands (32–34) and, through the activation of nuclear factor kappa-light-chain-enhancer of activated B cells (NF-κB) (35), may induce the expression of genes that encode pro-IL-1β, pro-IL-18, and NLRP3 (36, 37).

After this phase, in the activation step, a second signal, such as ATP, ion fluxes (K+, Ca++, Cl−) (28), β-amyloids, particles (such as uric acid, silica, aluminum), reactive oxygen species (ROS), and dysfunctional organelles, is required to form and assembly the inflammasome (10, 38–40).

Inflammasome activation may be regulated by post-translational modifications. For example, ATP and TLR-4 may activate the deubiquitination of NLRP3 in macrophages (41) by the enzyme BRCA1/BRCA2-containing complex subunit 3 (BRCC3) (42) and c-Jun N-terminal kinase (JNK1)-mediated NLRP3 S194 phosphorylation may induce NLRP3 deubiquitination with a subsequent inflammasome assembly (43).

Direct and indirect mechanisms of epigenetic regulation may activate the inflammasome. Wang et al. reported that overexpression of miR-377, through activation of the p38 MAPK/TXNIP/NLRP3 inflammasome pathway, was able to promote oxidative stress in diabetic nephropathy and prevent a severe glomerular podocyte inflammation (44). Other authors, reported that miR-711 suppressed NLRP3 expression by inhibiting FADD and NFkB (45), whereas miR-22, miR-30e and miR-223 by targeting directly the 3’UTR of NLRP3 gene (46–48).

Wu et al., showed that the NLRP3 inflammasome enhanced the chronic deleterious effect of hypoxia on proximal tubular cells and that miR-155, a positive-regulator of NLRP3 signals by inhibiting the targeted FOXO3a gene, could facilitate this biological effect (49).

These findings confirm previous results which showed that in tubular renal cells, the overexpression of miR-155 promotes upregulation of caspase-1, IL-1β, and IL-18, whereas knockdown of miR-155 attenuated the inflammatory cell death, suggested a potential use of the anti-miR-155 as preventive strategy against pyroptosis (50).

However, all these findings should be further studied and validated before being translated in the clinic.

## 4. Non-canonical activation of the NLRP3 inflammasome

The non-canonical activation of the NLRP3 inflammasome is caspase-1 independent and is mediated by caspase-4 and caspase-5 in humans and caspase-11 in mice (24).

These non-canonical caspases interact directly with LPS (51) and can activate both the NLRP3 inflammasome for cytokine production and GSDMD to mediate pyroptosis (11, 52–55).

Caspase-4, like caspase-1, may cleave GSDMD and pro-IL-18, but it seems not able to cleave IL-1β. Instead, caspase-5 cleaves GSDMD, but it has a very weak pro-cytokine activity (56), and caspase-11 has a proteolytic activity limited to GSDMD only (57).

In addition, caspase-8 may activate both canonical and non-canonical NLRP3 inflammasome pathways (33), but this effect needs to be better elucidated.

## 5. Inflammasomes in ischemia/reperfusion injury during kidney transplantation

Ischemia–reperfusion injury (IRI) is an almost inevitable process occurring after kidney transplantation, which includes a wide range of biological insults caused by the initial transient surgical warm ischemia followed by the cold ischemic period due to hypothermal maintenance/preservation of the organ (58).

In many recipients (approximately 15–25%), this process may lead to delayed graft function (DGF), a condition of no post-transplant recovery of renal function that requires the maintenance of dialysis treatment to ensure patient survival. Moreover, IRI activates a complex and multi-factorial biological network that may trigger acute allograft rejection and the early onset of interstitial fibrosis and tubular atrophy (IFTA). All these events may accelerate graft loss (58, 59).

In this process, activation of the inflammatory and innate immune system, including the inflammasome pathway, has a primary role (60). NLRP1, caspase-1, and NLRP3 expression appeared significantly up-regulated in renal tubular epithelial cells after IRI (61–63) and their persistent over-expression was associated with tubular maladaptive repair, large inflammatory cell infiltration, and organ fibrosis (64). Moreover, NLRP3^−/−^ mice but not Asc^−/−^ or caspase-1^−/−^ mice were protected from IRI, suggesting a direct effect of NLRP3 on tubular epithelial cells leading to IRI, but independent of inflammasome formation (65). However, during IRI, other pathways may be involved in this large biological networking (63). Recently, Lavallard et al. clearly demonstrated a direct activation of the NLRP3 inflammasome by ROS in rat islets undergoing IRI and that this effect was not prevented by N-acetyl-L-cysteine or caspase-1 inhibitors (66).

RNA-sequencing analysis in human kidney tissues obtained before ischemia (considered as a normal condition), 15 min after hypoxia (ischemia), and 10 min of reperfusion revealed significant up-regulation of the inflammasome (NLRC4) and immune response-related pathways after reperfusion (67, 68) with high urinary IL-18 content (69, 70).

IRI may be aggravated by a long cold storage time and may have a further negative impact on “fragile” organs from Extended Criteria Donors (ECD), which include donors over the age of 60 years or donors over the age of 50 years with 2 of the following 3 items: (1) history of high blood pressure, (2) serum creatinine ≥1.5 mg/dL, and (3) death due to stroke, with increased risk for allograft outcome (71, 72). These marginal organs, under specific stimulations, are often prone to release higher levels of pro-inflammatory cytokines and DAMPS compared to those from the Standard Criteria Donor (SDC). This may accelerate inflammatory-related allograft alterations (73).

In rat model of kidney transplantation, prolonged cold storage (18 h) induced a higher decline in renal function and a significant increment of IL-1β and NLRP3 protein levels compared with 2 h cold storage (this effect was similar in both deceased cardiac death (DCD) and living donor models) (74).

Moreover, the expression of several genes involved in the TLR-4 and inflammasome pathways was significantly upregulated in pre-implantation biopsies of ECDs compared with SDC, suggesting their potential role as therapeutic targets for new pharmacological treatments finalized to slow-down the progression of chronic allograft damage in marginal organs (75).

A promising therapeutic strategy to minimize IRI in kidney transplantation involves multipotent adult progenitor cells (MAPC). The addition of these cells to the normothermic machine perfusion solution significantly improved urine output, decreased the expression of NGAL (a main injury biomarker) and IL-1β, and upregulated anti-inflammatory and pro-tolerogenic cytokines (including IL-10 and Indolamine-2,3-dioxygenase) compared with untreated organs (76).

Activation of the NLRP3 inflammasome in IRI is also mediated by the non-canonical pathway. IRI may induce a marked increase in the expression of caspase-11 in renal tissue with subsequent pannexin 1 (panx1) activation by catalytic cleavage. This may facilitate ATP release and, through stimulation/activation of the NLRP3 inflammasome, promote kidney injury (77, 78).

Therefore, pharmacological inhibition of the NLRP3 inflammasome may represent a valuable therapeutic target to antagonize IRI-induced allograft damage.

Early post-transplant administration of MCC950 (50 mg/kg, i.p), a specific NLRP3 inhibitor that blocks inflammasome oligomerization, reduced the activation of caspase-1 and the release of IL-1β in allograft of mice that underwent IRI and ameliorated the post-transplant functional recovery of these organs (79).

Remdesivir, a widely employed antiviral nucleotide prodrug against COVID-19 (80), appears to antagonize acute kidney injury (AKI) by inhibiting NLRP3 inflammasome activation via the repression of NFkB and MAPK pathways in LPS-activated macrophages (81).

Roxadustat, a hypoxia-inducible factor (HIF) prolyl-hydroxylase inhibitor, when administered to mice before IRI (at a dose of 10 mg/kg for 5 days) alleviated renal damage by down-regulating AIM2 inflammasome with a consequent enhancement of HIF stabilization and activation of CD73/adenosine signaling (82).

A protective effect was also demonstrated for hydrogen sulfide (H2S), produced by the kidney under physiological conditions to promote kidney excretion, regulate renin release, and increase ATP production. H2S appears to have a protective role in kidney diseases by regulating oxidative stress, inflammation, and the renin–angiotensin–aldosterone system (83). In an animal model of IRI, the administration of H2S prior to surgery exerted this positive effect, at least partially, by Nrf2-mediated NLRP3 inflammasome inhibition (62).

Another drug with an inhibitory effect on the NLRP3 inflammasome is dexmedetomidine (DEX), a selective a2-adrenoreceptor agonist that regulates hemodynamics by reducing sympathetic tone, decreasing inflammatory response, inhibiting renin release, increasing glomerular filtration rate, and increasing secretion of sodium and water by the kidneys (84). Several animal studies have reported antioxidant, anti-apoptosis, and anti-inflammatory effects of DEX (85, 86).

Administration of DEX (30 μg/kg) 30 min before intraperitoneal injection of LPS in a mouse model of AKI attenuated renal injury by enhancing autophagy via the alpha2-adrenoreceptor/AMPK/mTOR pathway, which inhibits the NLRP3 inflammasome (87). This protective effect could explain the reduced incidence of DGF found in patients who received perioperative DEX during kidney transplantation (88).

GSK1070806, a humanized IgG1 antibody that binds and neutralizes the function of mature IL-18, has also been tested for the prevention of DGF in patients with DCD transplantation. However, administration of a single dose of this agent (3 mg/kg) just before kidney allograft reperfusion did not prevent this complication (89).

## 6. Inflammasomes in acute graft rejection

Acute rejection is a major complication of the allograft that could also occur very early post-transplantation and have a significant negative impact on graft survival. This condition, which could be triggered by humoral or T cellular immune dysfunctional regulation, has a complex and multi-factorial pathogenesis and a large involvement of the immune system.

Based on a few literature reports, inflammasomes may be involved in the onset and development of T-cell-mediated rejection (TCMR) and antibody-mediated rejection (ABMR).

Tejada et al., using a transcriptomics-based approach, revealed a significant enhancement of the AIM2 inflammasome in both T-cell- and antibody-mediated rejected allografts, even if the association was stronger for the former (90). These data were in line with those published by Venner et al. (91), which demonstrated only a small association between the AIM2 transcript and ABMR in a large biopsy transcriptomic analysis (703 biopsies) (92).

Asgari et al. showed that inflammasome activation could be induced by complement pathway mediators over-expressed during the rejection episode. In particular, C3a generated during inflammation engaged C3aR, increasing extracellular ATP, which, in turn, through P2X7 receptor signaling, activated inflammasome and IL-1β production by monocytes (93).

Moreover, donor-derived cell-free DNA (dd-cfDNA), deoxyribonucleic acid fragments that are released into the blood primarily during allograft rejection (94), could activate the inflammasome pathway (95). The binding of cfDNA to DNA sensor cyclic guanosine monophosphate (GMP)-adenosine monophosphate (AMP) synthase (cGAS) induces the synthesis of 2′3′ cyclic GMP–AMP (cGAMP), which activates stimulator of interferon genes protein (STING) and, in turn, the transcription of inflammatory genes via phosphorylation and activation of IRF3 (96). The cyclic dinucleotides produced by cGAS can also activate the NLRP3 inflammasome (97), and dd-cfDNA may directly activate the AIM2 inflammasome by binding to the HIN domain. However, additional studies are required to assess this important biological association.

Dessing et al., then, analyzing several NLRP3 SNPs in >1,200 matched donors and recipients, revealed that the NLRP3 gain-of-function SNP rs35829419 in donors was associated with an increased risk of acute rejection, whereas the NLRP3 loss-of-function SNP rs6672995 in recipients was associated with a reduced risk of rejection, particularly within the first year after transplantation (98). Unfortunately, the authors did not measure the specific production of inflammasome-related cytokines and did not clearly discriminate between cellular and humoral allograft rejection.

Finally, the administration of MCC950 and DEX, as in IRI, also demonstrated a significant protective effect against acute allograft rejection (79, 88), but with no real impact on short- and long-term allograft function (88). Nevertheless, the absence of large prospective studies and clinical trials on kidney transplantation makes it difficult to draw definitive conclusions.

## 7. Inflammasomes in viral and bacterial infections

Infections are frequent comorbidities in kidney transplant recipients that can directly or indirectly impact graft and patient survival. Although characterized by specific pathogenetic and biological mechanisms, these clinical complications may activate inflammation and, in some cases, the inflammasome pathway.

### 7.1. Viral infections

Epstein–Barr virus (EBV): it belongs to the human gamma herpesvirus family, with a seroprevalence of more than 90% in adults, and mainly infects B lymphocytes, epithelial cells, T lymphocytes, and NK cells (99).

In kidney transplant recipients, acute and reactivation of latent EBV infection may induce severe clinical complications, including the onset of a post-transplantation lymphoproliferative disorder (PTLD) (100–102), which may dramatically impact graft and patient survival.

Interestingly, the inflammasome pathway may play a role in maintaining the virus in a latent form. In fact, in EBV latent-containing B cells, Interferon Gamma Inducible Protein 16 (IFI16) recognizes the viral genome and, after recruitment of ASC and procaspase-1, forms an inflammasome complex, which moves into the cytoplasm, activates caspase-1, and cleaves pro-IL-1β, pro-IL-18, and pro-IL-33 into their mature forms (103). IFI16 and cytokines are subsequently sorted and released outside the cells via exosomes, representing a potential strategy to facilitate EBV persistence (103).

Another mechanism that continues viral latency in B lymphocytes is mediated by a physical interaction between IFI16 and the core constitutive heterochromatin machinery (KAP1 and SZF1), which silences the key EBV lytic switch protein (104).

Nevertheless, some authors have recently suggested that inflammasome pathways may also be involved in the EBV primary infection-related biological network. Lytic triggers, such as Histone deacetylase inhibitor (HDACi), DNA methyltransferase (DNMT) inhibitors, and Ig cross-linking, may induce caspase-1 activation through the thioredoxin-interacting protein (TXNIP)-NLRP3 inflammasome pathway, leading to partial loss of KAP1/TRIM28, a barrier to EBV lytic cycle entry (105, 106). However, all these observations should be confirmed in larger research and clinical studies.

The Human Herpesvirus-8 (HHV-8), also called Kaposi sarcoma Herpesvirus (KSHV), belongs to the family of DNA viruses Herpeseviridae and may be responsible for the onset/development of post-transplant cancer with single or multiple lesions on mucosal surfaces, including the skin, lungs, gastrointestinal tract, and lymphoid tissue (107). It can infect several different cell types, including endothelial cells, B cells, epithelial cells, dendritic cells, monocytes, and fibroblasts (108, 109).

The virus binds to several host cell surface receptors, such as integrins (including α3β1, αVβ5, and αVβ3), the cystine–glutamate transporter xCT, heparan sulfate, and the tyrosine protein kinase receptor EPHA2, and induces a signal transduction cascade, which results in cellular changes that allow the virus to enter the cell and traffic within the cytoplasm (110, 111).

Viral entry results in the delivery of the virion capsid into the cytoplasm, followed by its uncoating and the delivery of the HHV-8 genome into the nucleus, where it remains as an episome after circularization. Subsequently, the virus becomes latent or undergoes sporadic bouts of lytic reactivation during its lifecycle (108, 109).

Cellular entry or reactivation of virus activates an immune response via TLRs (TLR-3, TLR-4, and TLR-9) (112–114), retinoic acid-inducible gene I protein (RIG-I)-like receptors (RLRs), NLRs (NLRP1 and NLRP3) (115), ALRs and cyclic GMP-AMP synthase (cGAS)–STING (116).

The activation of these biological elements leads to the induction of type I interferon and NLR-dependent inflammasome pathways. However, Orf63, an HHV-8 tegument protein, with high sequence similarity to NLRP1, inhibits NLRP1 and NLRP3 inflammasomes by disrupting the association of NLRP1 and NLRP3 with ASC or procaspase-1 (115). Because Orf63 is a component of the tegument, it is released into the cytoplasm at the onset of primary infection, inhibiting host immunity.

Likewise, SOX protein, encoded by HHV-8 ORF37, with endo- and exo-nuclease activity for degrading cellular mRNA and processing the viral DNA genome, suppresses AIM2 inflammasome activation during the HHV-8 lytic cycle by disrupting AIM2: dsDNA polymerization and ASC recruitment and oligomerization (117).

Thus, targeting viral inhibitors of inflammasomes represents a therapeutic strategy for the treatment of viral infection.

BK polyomavirus (BKV): is a double-stranded DNA virus belonging to Polyomaviridae family and is highly prevalent in humans with an incidence of more than 80% in the general population (118). In immunocompetent individuals, primary BK virus infection is generally asymptomatic or results in mild symptoms, but in kidney transplantation, its reactivation in the tubular renal epithelial cells may induce cytotoxicity and allograft dysfunction (119).

Ribeiro et al., found that the inflammasome activator TLR-3 was upregulated in the tubule-interstitial allograft compartment of patients with polyomavirus-associated nephropathy (PVAN) along with IL-1β and IL-18 compared with acute rejection and pre-transplant donor biopsies (controls) (120, 121). However, surprisingly, the *in vitro* part of the study did not confirm the activation of the NLRP3 or AIM2 inflammasome pathways (120).

Severe acute respiratory syndrome (SARS) coronavirus 2 (SARS-CoV-2): is a positive-sense single-stranded RNA virus belonging to the coronavirus family responsible for coronavirus disease 2019 (COVID-19). This virus, which activates the NLRP3 inflammasome through the accessory protein viroporin E (122), could exacerbate the inflammatory machinery in infected kidney transplant recipients, leading to severe clinical complications and partly justify the positive effects of anti-inflammatory therapies in these fragile patients (123, 124).

These results, even if they did not allow to draw definitive conclusions, underline the necessity to study these pathways in kidney transplant patients.

### 7.2. Bacterial infections

Although inflammation plays a major role in urinary tract infections, only one study has clearly described the impact of the inflammasome in these common post-transplant complications.

It has been demonstrated that recombinant purified *E. coli* α-hemolysin caused deubiquitination, oligomerization, and activation of the NLRP3 inflammasome in response to K+ concentration perturbations in THP-1 macrophages. This led to mitochondrial dysfunction, and activation of the immune response, resulting in cell death (125). Additional studies are required to better address this important topic.

## 8. Inflammasome in recurrence of glomerulonephritis after kidney transplantation: a target to be explored in future

Glomerulonephritis (GNs), the fourth most common cause of allograft loss, are major clinical problems occurring in the post-transplant period. Most patients affected by these conditions may experience a significant reduction in allograft survival and develop severe systemic alterations (nephrotic and nephritic syndrome, acute allograft dysfunction, accelerated development of chronic allograft nephropathy) (126).

However, although no specific studies have been published regarding the impact of inflammasomes on the pathogenesis of recurrent glomerulonephritis (rGNs) after kidney transplantation, we can postulate their involvement in these disorders (127). In particular, hyperactivation of the NLRP3 inflammasome in infiltrating macrophages and podocytes (128, 129) and in serum of IgAN nephropathy (as demonstrated by high circulating level of IL-18 and IL-1β) is associated with a fast progression of this disease (127, 130).

In patients with focal segmental glomerulosclerosis (FSGS), necroptosis, a pro-inflammatory lytic form of programmed cell death activated in podocytes, activates the NLRP3 inflammasome pathway. As reported by Hu et al., the inhibition of necroptosis in adriamycin (ADR)-induced nephropathy, through the inhibition of this inflammasome in podocytes attenuates proteinuria levels and kidney histological damage (131).

In patients with ANCA-associated vasculitis (AAV), the serum level of IL-18 was elevated (132) and the tissue protein content of NOD2, NLRP3, and NLRC5 was higher than that in healthy controls, demonstrating the role of the inflammasome in these disorders. In addition, kidney tissue expression levels of NOD2 and NLRC5 were significantly correlated with the severity of renal lesions in AAV (133).

Therefore, despite the role of the inflammasomes in the pathogenesis of GNs in the native kidney, the impact of these biological pathways in the post-transplant recurrence should be addressed with specific research projects.

## 9. Conclusion

Our paper reveals the great interest of the transplant community in understanding the pathophysiological role of inflammasomes in kidney transplantation. Although the role of these biological pathways has been well elucidated in several native kidney disorders (134), few data regarding their direct contribution to allograft and systemic post-transplant complications are available (Table 1). Additionally, it underlines that a deep comprehension of the contribution of the inflammasomes in the biological machinery associated with acute and chronic allograft alterations may facilitate the discovery of novel previously unrecognized therapeutic targets, the identification of early diagnostic and prognostic biomarkers, and accelerate the initiation of clinical studies/trials involving inflammasome regulators (e.g., MCC950) finalized to slow down the progression of chronic allograft alterations and allograft functional failure. In this context, innovative biomolecular research strategies (including the high-throughput omics technologies) and an integrated/multi-disciplinary collaborative approach, which facilitate the interaction between different professionals (including molecular biologists, clinicians, bioinformaticians), could help accelerate this discovery process.

**Table 1:** Main studies focusing on the role of inflammasome pathways in clinical complications after kidney transplantation.

Clinical complication	Experimental model	Results	References
IRI	Murine model of kidney IRI	NLRC4 inflammasome was up-regulated in kidney tissue and macrophages of mice underwent IRI	(68)
	RNA-sequencing in human kidney tissues from normal (pre-ischemia), 15 min after hypoxia (ischemia), and 10 min of reperfusion	NLRC4 gene expression was up-regulated after reperfusion and not during ischemia	(67)
	Analysis of expression of genes involved in TRL4 pathway in kidney biopsies from Extended-Criteria Donors (ECD) and Standard Criteria Donor (SDC)	In pre-implantation kidney biopsies from ECDs the expression of genes involved in TLR4 and inflammasome pathways (NLRP3, CASP1, and IL-1β genes) was enhanced compared to kidney biopsies from SDC	(75)
	Renal tubular cells (RTECs) were subjected to cold storage and rewarming. Kidneys from wild-type or Casp1^−/−^ mice were subjected to cold ischemia (CI) for 30 min and then transplanted into wild-type recipients (CI + Txp)	NLRP1 and Caspase-1 expression was significantly enhanced in renal tubular epithelial cells after IRI and in wild-type kidneys subjected to CI + Txp into wild-type recipients compared to kidneys transplanted from Casp1^−/−^ donors into wild-type recipients	(63)
	Animal model of IRI in wild-type mice and mice deficient in components of the Nlrp3 inflammasome (Nlrp3^−/−^ and Asc^−/−^ mice)	NLRP3^−/−^ mice but not Asc^−/−^ or caspase-1^−/−^ mice were protected from IRI, suggesting a direct effect of NLRP3 on tubular epithelial cells leading to IRI, but independent of inflammasome formation	(66)
	Animal model of IRI in WT and NLRP3^−/−^ mice	Damaged mitochondria in renal IRI were a major source of ROS contributing to NLRP3 inflammasome activation by direct TXNIP-NLRP3 interactions	(61)
	Wild-type and Nrf2-KO mice underwent renal IRI. MCC950 was injected intraperitoneally daily for 14 days before surgery (I/R + MCC950 group). NaHS (50 μmol/kg) was injected intraperitoneally before surgery (I/R + NaHS group)	Renal IRI-induced activation of the NLRP3 inflammasome pathway in WT and Nrf2-ko mice. Treatment with MCC950 downregulated NLRP3, ASC, caspase-1 and IL-1β expression levels following renal IRI. NaHS decreased NLRP3 inflammasome activation via the Nrf2 signaling pathway	(62)
	Animal model of IRI. The mice underwent mild and severe IRI induced by 15 and 25 min of renal ischemic duration, respectively. Short- and long-term outcomes were evaluated at 2 and 28 days after surgery	In severe IRI, there was a long-term high level of NLRP3 in serum and urine. NLRP3 overexpression was mainly distributed in the abnormal tubules surrounded by inflammatory infiltrates and fibrosis, which indicated maladaptive repair. Renal NLRP3 overexpression correlates with infiltrating macrophages and fibrosis	(64)
	In a cohort of 91 patients serial urine samples were collected for 3 days after kidney transplantation	Elevated urinary IL-18 levels predicted the need for dialysis within the first week of kidney transplantation and 3-mo recovery of graft function	(69)
	Transcriptional analysis of kidney biopsies from patients with DGF	Up-regulation of NLRC4, IFNγ and IFNγ-inducible targets OAS2 and CXCL10 suggested inflammasome activation in allografts with DGF	(70)
	Donation of circulatory death (DCD) kidneys and living donor (LD) kidneys of male SD rats were preserved in UW solution at 4°C for 2 h or 18 h and then transplanted into syngeneic recipients	Prolonged cold storage (18 h) induced a higher decline in renal function and a significant increment of IL-1β and NLRP3 protein compared to 2 h cold storage (this effect was similar in both DCD and LD models)	(74)
	Animal model of IRI in Casp-11^−/−^ and wild type mice	IRI markedly increased caspase-11 expression and pannexin 1 (panx1) cleavage in the kidneys accompanied by NLRP3 inflammasome activation in wild-type mice. In Casp-11^−/−^ mice, I/R-induced panx1 cleavage, NLRP3 inflammasome activation, renal functional deterioration, and tubular morphological changes were significantly attenuated. The cleavage of panx1 by upregulated casp-11 is involved in facilitating ATP release and NLRP3 inflammasome activation in IRI	(77)
	Rat model of renal IRI	Pyroptosis-related proteins (casp-1, casp-11, and IL-1β) were significantly increased after 6 h of renal IRI and peaked 12 h after injury. Enhanced pyroptosis was accompanied by elevated renal structural and functional injury. Up-regulation of endoplasmic reticulum (ER) stress biomarker (CHOP) preceded the incidence of pyroptosis showing that the CHOP-casp-11 pathway is crucial for IRI-related renal pyroptosis	(78)
	Rat model of renal transplantation. MCC950 was injected into animals (50 mg/kg, i.p) twice per week after surgery for 7 days	MCC950 reduced the activation of casp-1, alleviated the release of IL-1β, attenuated the active form of GSDMD and improved graft functional recovery on the 7th day after Tx	(79)
	Mice were administered Remdesivir (RDV) (25 mg/kg every 12 h for 7 days) by subcutaneous injection. After 7 days of continuous subcutaneous injection, mice in the LPS and RDV + LPS groups were i.p. injected with 10 mg/kg LPS to induce AKI	Remdesivir suppressed NLRP3 inflammasome activation through inhibition of NFkB and MAPK pathway, thereby reducing inflammation-induced renal damage and improving the recovery of renal function after AKI	(81)
	Roxadustat was administered (10 mg/kg/d i.p.) in mice for 5 days before IRI	Roxadustat attenuated renal tubular injury in the IRI mice model by suppressing the activation of AIM2 inflammasome and the increase in the CD73 pathway	(82)
	In a cohort of 780 patients who underwent kidney transplantations, 315 received intravenous dexmedetomidine (DEX) infusion (0.24–0.6 ug/kg/h) during surgery, and 465 did not	DEX use significantly decreased DGF, risk of infection, risk of acute rejection, overall complications, and length of hospital stay in patients who underwent kidney transplantation	(88)
	Rat model of AKI by intraperitoneal injection of 10 mg/kg LPS. The rats received intraperitoneal injections of DEX (30 μg/kg) 30 min before an intraperitoneal injection of LPS	DEX significantly attenuated renal injury in AKI by decreasing activation of the NLRP3 inflammasome and expression of IL-1β and IL-18. In addition, DEX could significantly enhance autophagy via the alpha2-adrenoreceptor/AMPK/mTOR pathway, which inhibits the NLRP3 inflammasome	(87)
Acute rejection	Transcriptome analysis comparing kidney biopsies from patients with acute rejection and those with stable grafts	Rejected grafts showed inflammasome enhancement and, in particular, AIM2 expression was positively correlated with T-cell activation and negatively correlated with oxidative phosphorylation metabolism	(90)
	Monocytes isolated from healthy subjects and biopsy samples from patients with acute rejection	Kidney transplant biopsies from patients with acute rejection were characterized by local generation of C3a and monocytes and Th17 cell infiltration. The authors proposed a model in which C3a generated during inflammation engaged C3aR, increasing the extracellular ATP, which, in turn, through P2X7 receptor signaling, activated inflammasome and IL-1β production leading to enhanced Th17 responses that contribute to acute rejection	(93)
	NLRP3 single nucleotide polymorphisms (SNPs) in >1,200 matched donors and recipients	NLRP3 gain-of-function SNP rs35829419 in donors was associated with an increased risk of acute rejection, whereas NLRP3 loss-of-function SNP rs6672995 in recipients was associated with a reduced risk of rejection	(98)
Infection Epstein–Barr virus (EBV)	EBV latency I Raji cells	In EBV latent containing B cells Interferon Gamma Inducible Protein 16 (IFI16) recognizes the viral genome and, after recruitment of ASC and procaspase-1, forms an inflammasome complex, which migrates in the cytoplasm, activates caspase-1, and cleaves pro-IL-1β, pro-IL-18, and pro-IL-33 into their mature forms. IFI16 and cytokines are subsequently sorted and released to the exterior of the cells via exosomes, which represents a potential strategy to facilitate EBV persistence	(103)
	EBV-positive Burkitt lymphoma (BL) cell lines	IFI16 physically partners with the core constitutive heterochromatin machinery (KAP1 and SZF1) to silence the key EBV lytic switch protein, thereby ensuring continued viral latency in B lymphocytes	(104)
	EBV-positive endemic BL cell lines	Lytic triggers, such as Histone deacetylase inhibitor (HDACi), DNA methyltransferase (DNMT) inhibitors, and Ig cross-linking, may induce caspase-1 activation through the thioredoxin-interacting protein (TXNIP)-NLRP3 inflammasome pathway, leading to partial loss of KAP1/TRIM28	(106)
Human Herpesvirus-8 (HHV-8)	THP-1 cells stably expressing Orf63	Cellular entry or reactivation of virus also activates an immune response via NLRs. Orf63, an HHV-8 tegument protein, with high sequence similarity to NLRP1, inhibits NLRP1 and NLRP3 inflammasomes by disrupting the association of NLRP1 and NLRP3 with ASC or procaspase-1	(115)
	KSHV-positive and latently infected cell lines	SOX protein encoded by HHV-8 ORF37 suppresses AIM2 inflammasome activation during the HHV-8 lytic cycle by disrupting AIM2: dsDNA polymerization and ASC recruitment and oligomerization	(117)
BK polyomavirus (BKV)	Human renal biopsies from acute rejection and PVAN	The inflammasome activator TLR-3 was upregulated in the tubule-interstitial allograft compartment of patients with PVAN along with IL-1β and IL-18 compared with acute rejection and pre-transplant donor biopsies (controls)	(120)
Bacterial infection	THP-1 derived macrophages were incubated with HlyA and proHlyA along (220 ng/mL) with control (mock/without any stimulation) for 2 h	Recombinant purified *E. coli* α-hemolysin caused deubiquitination, oligomerization, and activation of the NLRP3 inflammasome in response to K+ concentration perturbations in THP-1 macrophages. This led to mitochondrial dysfunction and activation of the immune response, resulting in cell death	(125)

## Author contributions

SG: Writing – original draft. DL: Writing – original draft. GS: Writing – review & editing. AP: Writing – review & editing. GZ: Conceptualization, Funding acquisition, Writing – original draft.
